# Nanoparticles-Delivered Circular RNA Strategy as a Novel Antitumor Approach

**DOI:** 10.3390/ijms25168934

**Published:** 2024-08-16

**Authors:** Luisa Racca, Elisabetta Liuzzi, Simona Comparato, Giorgia Giordano, Ymera Pignochino

**Affiliations:** 1 Department of Clinical and Biological Sciences, University of Turin, 10043 Orbassano, Italy; luisa.racca@uniupo.it (L.R.); simona.comparato@unito.it (S.C.); 2 Center for Translational Research on Allergic and Autoimmune Diseases (CAAD), Università del Piemonte Orientale, 28100 Novara, Italy; 3 Department of Translational Medicine, Università del Piemonte Orientale, 28100 Novara, Italy; 4 Princess Máxima Center for Pediatric Oncology, 3584 CS Utrecht, The Netherlands; e.liuzzi@prinsesmaximacentrum.nl; 5 Candiolo Cancer Institute, FPO-IRCCS, 10060 Candiolo, Italy; giorgia.giordano@ircc.it; 6 Department of Oncology, University of Turin, 10060 Turin, Italy

**Keywords:** circRNA, nanomedicine, theranostics, circRNA-based therapeutic, nanoparticles, nanomaterial, gene therapy, drug delivery, cancer therapy

## Abstract

Anticancer therapy urgently needs the development of novel strategies. An innovative molecular target is represented by circular RNAs (circRNAs), single-strand RNA molecules with the 5′ and 3′ ends joined, characterized by a high stability. Although circRNA properties and biological functions have only been partially elucidated, their relationship and involvement in the onset and progression of cancer have emerged. Specific targeting of circRNAs may be obtained with antisense oligonucleotides and silencing RNAs. Nanotechnology is at the forefront of research for perfecting their delivery. Continuous efforts have been made to develop novel nanoparticles (NPs) and improve their performance, materials, and properties regarding biocompatibility and targeting capabilities. Applications in various fields, from imaging to gene therapy, have been explored. This review sums up the smart strategies developed to directly target circRNAs with the fruitful application of NPs in this context.

## 1. Introduction

CircRNAs are involved in many hallmarks of cancer (e.g., cell proliferation, immune evasion, dedifferentiation, angiogenesis, invasion, metastasis, and drug resistance). They are intervein in important cancer signaling pathways, including Wnt/β-catenin signaling, mitogen-activated protein kinase (MAPK)/Extracellular regulated-kinase (ERK) and Phosphatase and Tensin Homolog (PTEN)/Phosphatidylinositol-4,5-Bisphosphate 3-Kinase (PIK3)/AKT Serine/Threonine Kinase 1 pathways, p53, and K-Ras [1,2,3,4,5]. CircRNAs have been directly implicated in the transcription modulation of both oncogenes and tumor suppressors, and they can sponge microRNAs (miRNAs) and regulate RNA-binding proteins (RBPs) involved in cancer pathways [6,7,8,9,10]. The precise molecular targeting of key circRNAs is an attractive strategy for developing novel anticancer therapy. Small interfering RNAs (siRNAs) and antisense oligonucleotides (ASOs) are the specific tools for the degradation or modulation of oncogenic circRNA functions. Moreover, overexpression vectors or direct tumor-suppressor circRNAs vehiculation can be achieved, and nanotechnology enables the optimization of both the specific targeting and delivery [11,12,13,14].

## 2. Circular RNAs

**Figure 1:**
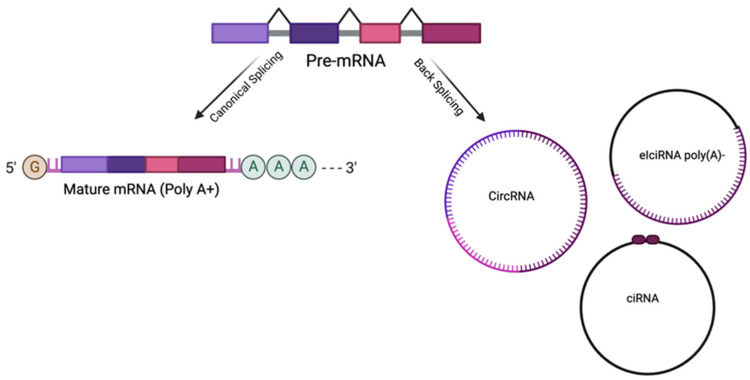
Schematic representation of canonical and back-splicing events of a single-strand pre-mRNA molecule (created with Biorender.com).

CircRNAs are single-strand RNA molecules formed by an alternative splicing process named back-splicing, where the 3′ end and the 5′ end of a pre-mRNA are joined through a phosphodiester bond, forming a circular structure and lacking the Poly(A) and the methylguanosine Cap (Figure 1). Back-splicing is advantaged when the pre-mRNA canonical processing is slowed down [15,16,17].

CircRNAs are made with one or more exons (exonic circRNAs, ecircRNA), exons and introns (exon–intron circRNAs, EIciRNAs), or one or more introns (intronic circRNAs, ciRNAs) [6,11,18]. CircRNAs coming from exon circularization are normally localized in the cytoplasm, whereas the intron derivatives (ciRNAs, EIciRNAs) are retained in the nucleus, where they are involved in the modulation of gene expression mechanisms [19,20]. However, exceptionally, some ecircRNAs have been observed in the nucleus, where they are involved in protein retention or protein recruitment to chromatin [21,22]. Some circRNAs are also present in mitochondria [23]. Interestingly, circRNAs can leave the intracellular spaces transported by extracellular vesicles (EVs) for specific functions in cell-to-cell communication and, thus, be found in biofluids such as urine and plasma [24,25,26]. 

### 2.1. CircRNA Biological Role

The biological role of circRNAs is still under wide investigation. Four main functions are known so far: transcription modulation, miRNA sponging, interaction with RBPs, and translation of peptides. 

#### 2.1.1. Transcription Modulation

**Figure 2:**
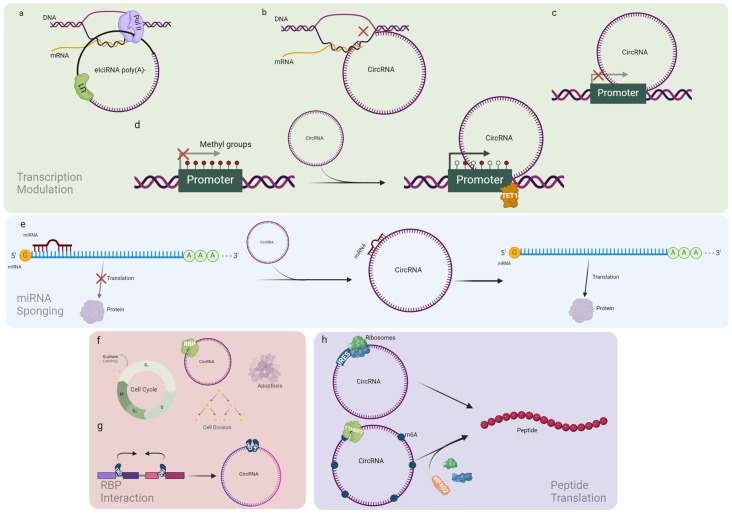
Schematic representation of circular RNA biological roles: (**a**) EiciRNA and ciRNA form a complex with U1 and small nuclear ribonucleoprotein (snRNP) inducing the interaction with RNA polymerase, enhancing gene expression; (**b**) circRNAs bind the host gene, pausing or ending its transcription; (**c**) circRNAs bind to the promoter preventing the association of the transcription complex; (**d**) host gene transcription promotion due to TET1-induced hypomethylation on CpG islands promoter, driven by circRNA binding; (**e**) miRNA sponging mechanism; (**f**) RBP interaction may alter several cell life aspects; (**g**) QKI’s dimerization enhancing circRNA biogenesis; (**h**) IRES-driven and m6A-driven peptide translation (created with Biorender.com).

Even though the circRNA-controlling process of transcription is still poorly characterized, some different mechanisms have been described: (i) interaction with the small nuclear ribonucleoprotein U1 in the nucleus, forming a complex bound to the RNA polymerase II and enhancing parental gene expression (Figure 2a); (ii) binding of the host gene to form a heteroduplex (RNA-DNA), pausing or ending the transcription (Figure 2b); promoter binding to limit the attachment of the transcription complex (Figure 2c); (iii) interaction with epigenetic factors, e.g., the demethylase enzyme TET1 inducing epigenetic silencing (Figure 2d) [21,27]. A further regulation occurs when one or more of the above-mentioned mechanisms directly affect the expression of transcription factors [28]. 

#### 2.1.2. miRNA Sponging

The most studied biological role of circRNAs is miRNA sponging. MiRNAs are indeed RNA molecules able to impair the translation of a targeted mRNA. According to the complementarity of the sequence, after binding to the mRNA target, an AGO2-mediated cleavage or a deadenylation of the 3′ end of the mRNA leads to mRNA degradation [29]. CircRNAs can interact with a miRNA, impairing its binding with the target mRNA, which, in turn, is translated (Figure 2e) [6,21]. 

#### 2.1.3. RBP Interaction

CircRNAs can also sponge RBPs, modulating their functions or sequestering them to specific cellular locations, leading to the modification of downstream pathways or gene expression [30,31]. This mechanism is still under intense investigation in several cellular and biological functions such as cell proliferation, cell cycle control, apoptosis, and specialized cellular functions (Figure 2f). As an example, here, we mention a circRNA derived from *Forkhead Box Protein O3* (circFoxo3) that directly interacts with the oncosuppressor p21 and the cyclin-dependent kinase 2 controlling cell cycle progression [32]. Another example is a circRNA derived from the Antisense Noncoding RNA in the *INK4* Locus (*circANRIL*) that binds the protein Pescadillo Ribosomal Biogenesis Factor 1 (PES1), controlling the maturation of ribosomal RNAs [33]. A further example is the circRNA originating from the second intron of the insulin gene (*Ci-Ins2*) that binds the TAR DNA Binding Protein, controlling insulin secretion [34]. Physiologically, RBPs are also involved directly in the biogenesis of circRNAs. This connection is mediated by the presence of a consensus motif frequently found on the flanking introns of the circRNAs recognized by the two monomers of the RBP Quaking (QKI) that bind the upstream and downstream flanking introns, respectively, of the future circRNA sequence, and their dimerization enhances the circularization of the RNA (Figure 2g) [31]. The modulation of circRNA biogenesis is implicated in several physiological and pathological cellular processes, i.e., cell proliferation, apoptosis, cell cycle control, and metabolism, as reviewed elsewhere [28].

#### 2.1.4. Peptide Translation

One of the most interesting but also less characterized circRNAs’ features is their ability to encode for a peptide. Indeed, circRNAs containing an open reading frame (ORF) and an internal ribosome entry site (IRES) can be translated by a cap-independent mechanism into circRNA-derived peptides. The lack of data concerning this property is due to the infrequency of IRES in eukaryotic transcriptomes, and additionally, less than 1.5% of circRNAs possess an IRES sequence, making the number of putative circRNA-derived peptides small. However, it was found that circRNAs are enriched in IRES-like short elements, i.e., AU-rich hexamers sequences allowing for cap-independent translation [35]. 

Meanwhile, peptide translation can also occur without an IRES sequence or IRES-like short elements. As an example, the methylation of the adenine nitrogen in position 6 (m6A), induced by the methyltransferase 3 and 14 enzymatic complex and regulated by m6A demethylase Fat mass and obesity-associated protein (FTO) [35,36], leads to the translation of the peptide. This is driven by recognizing the RBP YTH-domain family 3 that recruits the Eukaryotic Translation Initiation Factor 4 Gamma 2, and then the ribosomes (Figure 2h). 

Of course, even with IRES, IRES-like elements, or m6A modifications, only circRNAs containing an ATG start codon can be translated into a peptide.

However, the presence of the above-mentioned prerequisites in the circRNA sequence does not imply that it will be translated into a stable protein, because, for example, it could be rapidly degraded due to its inefficient folding [35].

### 2.2. CircRNAs and Cancer

**Figure 3:**
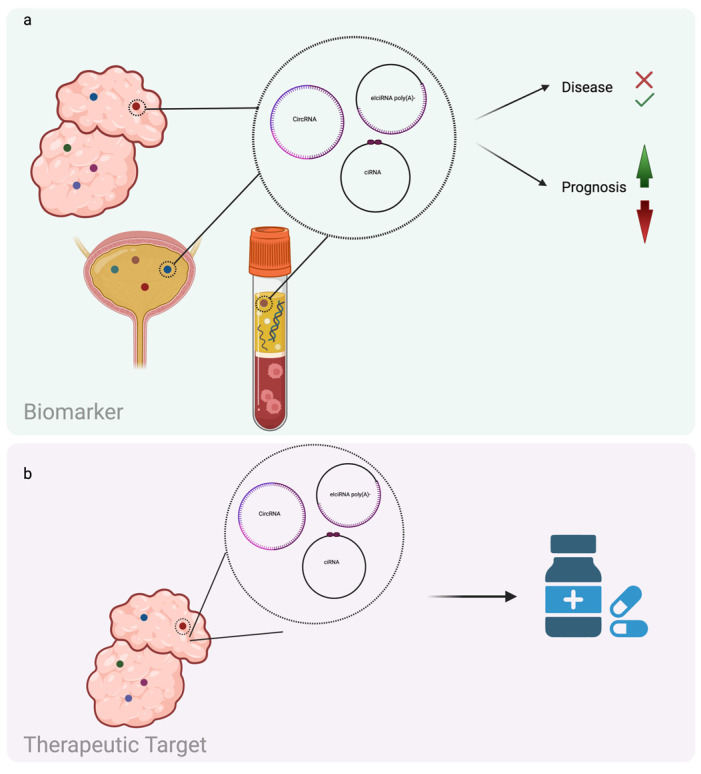
(**a**) Schematic representation of circRNAs present in tissues and biofluids, usable as diagnostic and prognostic biomarkers; (**b**) circRNAs in tissues exploitable as therapeutic targets (created with Biorender.com).

As mentioned above, circRNAs act in several aspects of cell life, and alterations of their level could be detrimental to cells, resulting in pathological conditions. A growing body of evidence has shown that circRNAs are involved in the maintenance of stem cell pluripotency and cellular differentiation, and their deregulation is implicated in cancer stemness features [37,38]. CircRNAs are involved in different aspects of cancer onset and progression [6,39]. The circRNAs can be present both in tissues and biofluids [40]. So, their secretion through EVs and their intrinsic stability in biofluids and tissues make them potentially good diagnostic and prognostic biomarkers (Figure 3) [41]. As an example, circRNAs are found to be differentially expressed in colorectal, liver, and lung cancer patients’ serum if compared to healthy donors, and the quantification of circRNAs in EVs gives rise to new insights into cancer progression and response to therapies [26,42]. The demonstration of circRNA’s functional role in cancer makes them also promising therapeutic targets (Figure 3).

Also, in the case of circRNA intervention in cancer, it is possible to distinguish their main molecular mechanisms. For example, miRNA and RBP sponging is the main mechanism of the circRNAs involved in the promotion, suppression, and resistance of breast cancer to therapy [43,44,45]. For instance, a circRNA derived from the gene of the DNA methyltransferase 1 (*circDNMT*) is upregulated in breast cancer cell lines and tissues and stimulates cancer cell autophagy through interaction, and the consequent nuclear translocation of the onco-suppressor p53 and the RBP Heterogeneous Nuclear Ribonucleoprotein D also known as Auf1 [44]. Other circRNAs may also be involved in response to drug treatment: a circRNA derived from *phosphoglycerate mutase family member 5* (*PGAM5*) mRNA is a determinant of tamoxifen resistance. Its depletion restores the sensitivity of tamoxifen-resistant breast cancer cells, and, therefore, the combination of tamoxifen and *sh-circPGAM5* treatment significantly reduces tumor growth in vivo. These effects are likely related to the *circPGAM5*/miR-876-3p/actinin Alpha 4 axis [46].

As mentioned above, the role of circRNAs in cell physiology varies widely, sometimes regulating malignant processes and sometimes possessing protective properties. An example of the latter is a circRNA derived from semaphorin A4B (*circSEMA4B*) and its potential protective role against breast cancer. This circRNA can be translated into the tumor-suppressing peptide SEMA4B-211aa, and so cancer cells try to counteract its activity by downregulating its expression. Specifically, SEMA4B-211aa binds to the regulatory subunit of the Phosphatidylinositol-4,5-Bisphosphate 3-Kinase (p85), inducing the formation of the p85-p110 complex and, thereby, impairing the phosphorylation of Akt and its downstream pro-tumorigenic signaling pathways [47]. So, inducing the overexpression of this circRNA could potentially produce positive outcomes in the treatment of this pathology.

Another circRNA with anti-tumor action is the one derived from the long non-coding RNA *Cerebellar Degeneration Related 1 antisense CDR1as* (*circCDR1as*). It is highly expressed in the brain and melanocytes, while it is downregulated in melanoma tissue. *CircCDR1as* interacts with the RBP Insulin-Like Growth Factor 2 mRNA Binding Protein 3 (IGF2BP3), modulating its downstream targets. The silencing of *circCDR1as* leads to an enriched expression of the IGF2BP3 downstream pro-metastatic targets, resulting in an increased invasion in vitro and metastasis formation in vivo [48]. One of the outputs from the circRNA-RBP interaction is the mRNA stability modulation. One other example of this mechanism is represented by a circRNA derived from the mRNA of the Zinc-finger protein 609 (*circZNF609*). It destabilizes the mRNA of Rac Family Small GTPase 1 (RAC1), a known pro-tumorigenic factor highly expressed in metastatic melanoma patients, associating with its RBP Fragile X Messenger Ribonucleoprotein 1, and resulting in RAC1 destabilization. To contrast its anti-tumor action, it is often downregulated in acral and cutaneous melanomas [49]. An example of pro-tumorigenic mRNA stabilization is represented by a circRNA derived from *Cell Division Cycle 45* mRNA (*circCDC45*), which stabilizes the mRNA of its host gene through the interaction with the RBP Eukaryotic Translation Initiation Factor 4A3 (EIF4A3). *CircCDC45* silencing led to CDC45 destabilization, increased apoptosis, decreased viability, and invasion in melanoma in vitro and in vivo assays [50]. The translation of circRNA into pro-oncogenic peptide was described in breast cancer. Thirty percent of the so-called triple-negative breast cancers expressed HER2-103, a small protein translated from the *Circular HER2* RNA (*circ-HER2*). Its expression is related to worse prognosis, and its knockdown inhibited cell proliferation, invasion, and tumorigenesis in vitro and in vivo. HER2-103 shared most of the same amino acid sequences of the linear form, HER2, which could be antagonized by the clinically approved Her2 blocking antibody Pertuzumab. It showed in vivo antitumor activity of circ-HER2/HER2-103 positive breast cancer cells [51]. Other examples of circRNA-derived peptides involved in cancer onset and progression have been exhaustively reviewed recently [52] 

## 3. Nanomedicine

The reported evidence shows that circRNAs could represent an intriguing therapeutic target, both to be blocked or enhanced, being involved in the development and progression of cancer or its control. Pro-tumoral circRNAs can be blocked by using silencing RNAs (siRNAs), short hairpin RNAs (shRNAs), or ASOs complementary to the back-splicing junction to avoid silencing of the corresponding linear counterpart. Tumor-suppressor circRNAs can be overexpressed by using expression vectors, i.e., plasmids or viral vectors, which are delivered to the target cells [53]. The efficient delivery of all these tools can be achieved by nanotechnology. In the last decades, the application of nanotechnology for the diagnosis, monitoring, and treatment of diseases, especially cancer, has emerged as an innovative approach called nanomedicine [54,55,56]. The number of investigations in this area has been steadily on the rise [57,58,59]. Great feasibility, scalability, and high cost-effectiveness are the winning advantages of these applications [60]. Although it is generally believed that this is a recent medical advance, it is reported that nanotechnologies have been used in the past, e.g., in India, Egypt, and China [61]. 

### 3.1. Nanomaterials and NPs

Based on dimensionality, nanomaterials are classified into four classes: 0D, 1D, 2D, and 3D. Nanoparticles (NPs) belong to the 0D (zero-dimensional) nanomaterials, which have their height, length, and width in the nanoscale range and are the most frequently cited in nanomedicine. On the other hand, 1D, 2D, and 3D (one-, two-, or three-dimensional nanomaterials) have one, two, or all dimensions outside the nanoscale. For example, nanotubes, nanofilms, and nanotube arrays are examples of 1D, 2D, and 3D nanomaterials, respectively [62]. It is worth noting that the latter class consists of objects that, as a bulk, exceed the dimensions of the nanoscale, but whose individual units, i.e., the nanotubes that form the array, fall within the dimensions of the nanoscale. 

NPs can be synthesized with different shapes, sizes, and compositions to obtain novel properties. 

**Figure 4:**
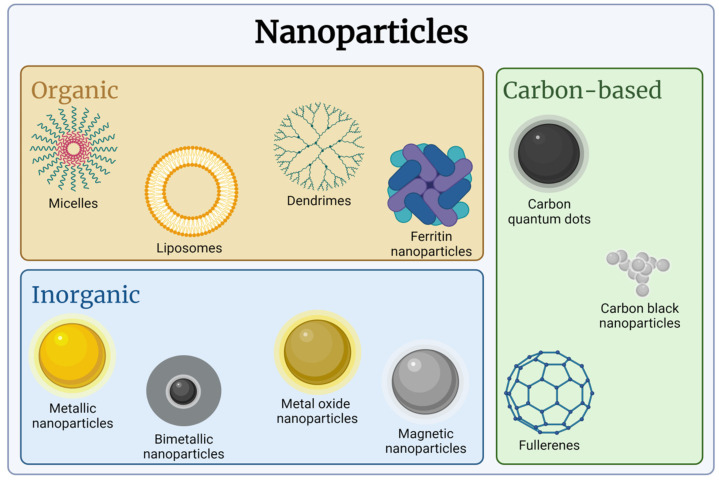
Nanoparticles’ classification based on their chemical composition (created with Biorender.com).

As shown in Figure 4, organic, inorganic, and carbon-based NPs are divided into several subcategories according to their chemical composition [62]. Organic NPs are synthesized from polymers, proteins, carbohydrates, lipids, and any other organic molecule [63]. Liposomes, micelles, and dendrimers are examples of this category, but there are many more. They have several advantages, such as low cytotoxicity and biocompatibility, which make them suitable for biological applications [62]. Liposomes are composed of a phospholipid bilayer [63], while micelles are formed by a single layer constituted by amphiphilic molecules differentially arranged to expose the hydrophilic or hydrophobic portion on the outside. Dendrimers instead are complex structures composed of several symmetric branches conjugated to a core and have several possible compositions [64]. Further examples of organic NPs are albumin or ferritin-based ones, which are NPs built with serum albumin or protein for iron storage ferritin. They are often cited for their extensive use in nanomedicine [65,66]. Liposomes have been extensively studied in the field of nanomedicine because they have an internal hole that allows the delivery of a payload, e.g., a drug, and are easy to prepare using already developed manufacturing syntheses [63]. In addition, cationic lipid NPs, solid lipid NPs, nanostructured lipid carriers, and nonlamellar lipid NPs are emerging as alternatives to liposomes because they are their “updated” version, with elaborate internal architecture and enhanced stability [67]. The considerable number of lipidic and polymeric NPs already approved by the FDA, however, confirms the clinical success of the organic NPs [68]. Two successful examples of nanotools in routine clinical use are Doxil, liposomes carrying the antineoplastic drug doxorubicin, and nab-paclitaxel, an albumin-based nanoformulation of taxane [69].

Inorganic NPs are composed of one or more inorganic compounds. They could indeed be made up of different materials and are classified according to their chemical composition and properties: metallic (gold and silver NPs), bimetallic (such as iron–cobalt and iron–nickel) metal oxides (such as titanium dioxide and zinc oxide NPs), and magnetic (such as iron oxide NPs) (Figure 4). The shape of these NPs may be different: they can have a solid morphology, e.g., spheres, polygons, stars, and others, or a mesoporous/hollow morphology, with cavities inside [70]. Each material has different physicochemical properties and, thus, can be exploited for medical purposes, but on the other hand, it is characterized by specific drawbacks. The reduction to the nanoscale indeed changes the toxicity profile of the material, and the small size allows for its direct interaction with cellular components, e.g., proteins, altering cell physiology and affecting their long-term biocompatibility [71,72]. For instance, zinc oxide NPs possess some intriguing piezoelectric, optical, and antibacterial properties among others and have been employed in several sectors for human health and anticancer purposes but are characterized by strong cytotoxicity due, for example, to the release of toxic ion species, and this is the reason why these NPs are often combined with other materials and/or coated with different envelopes to prevent their cytotoxicity [73,74]. Also, gold, which is inert in the bulk form, acquires other properties when reduced in size but, as in the case of zinc oxide, it could be easily modified and functionalized to prevent possible toxic effects, and, indeed, it is one of the most popular metallic NPs, finding several applications in the biomedical field [75].

Finally, the third class is represented by carbon-based NPs. Carbon quantum dots, carbon black NPs, and fullerenes are examples of carbon-based NPs (Figure 4). Carbon quantum dots are small NPs with a size below 10 nm. Carbon black NPs are aggregates of spherical NPs [76]. Fullerenes are hollow clusters of carbon atoms with sp2-hybridization, with a variable number of carbon atoms, even if the configuration with 60 carbon atoms is the most stable and commonly used. They are poorly soluble in water and, therefore, in biological fluids, and for this reason, they are usually modified to be more hydrophilic, allowing for their application for several purposes [77].

### 3.2. NPs in the Body Fluids and Coating Agents

In general, NPs should be designed and constructed keeping in mind that they need to interact with cells and body fluids before triggering their therapeutic or diagnostic action at the target site. NPs could cause serious side effects depending on the NP’s composition, size, shape, and surface charge [78]. For example, NPs have been reported to interact with erythrocytes and platelets, causing hemolysis and thrombus formation. Furthermore, their off-target action due, for example, to their degradation, could potentially also injure healthy cells and reduce their therapeutic efficacy. Moreover, the coating of NPs by the components of body fluids, forming the so-called “protein corona”, could alter their target action and colloidal and physicochemical properties, and even the immune system could contribute to their elimination, therefore vanishing their therapeutic or diagnostic action [79]. 

To preserve and enhance the efficacy of NP therapy, the first step is obviously dimension control because NPs smaller than 10 nm are exposed to renal clearance, while they are eliminated by phagocytes if the size exceeds 100 nm [71]. More importantly, NPs could be sterically stabilized, therefore avoiding their aggregation and direct contact with the biological fluids by the addition of stabilizing agents. One of the most popular strategies is the addition of the polymer poly (ethylene glycol), which improves stability, gives stealth properties, and fights the opsonization process, thus reducing the uptake by macrophages [79]. The coating with phospholipid bilayers, creating a sort of liposome with NPs inside, to chemically prevent their degradation and agglomeration is also frequently reported [80,81]. Another possibility is to use biocoatings, i.e., coatings derived directly from cell components and biofluids, as EVs. In this context, several investigations have been conducted in recent years with promising results, paving the way for the adoption of such nanoconstructs in future clinical practice [82].

However, in all cases mentioned above, the addition of specific ligands, e.g., folic acid particularly necessary for the tumor, or specific antibodies directed against tumor cells could improve the targeting of NPs [83,84].

### 3.3. EVs as a Natural and Pathological Delivery System

EVs are employed as coating agents of other NPs, or as NPs themselves, even if their variable dimensions (up to 1000 nm) make them often excluded from the list of “conventional” organic NPs. The term EVs includes exosomes, ectosomes, microvesicles, membrane vesicles, and apoptotic bodies present in various body fluids, e.g., milk and blood. They have distinct roles in cell physiology, being involved in cell–cell communication. From the chemical point of view, they could be described as liposomes with a more complex structure because they are constituted by a phospholipid bilayer enriched with other lipids, such as cholesterol, and proteins, such as several tetraspanins, delivering various cargoes [85,86]. It is worth mentioning that the composition of the membranes and the cargo vary according to the type of vesicles and cells producing EVs. Also, dimensions vary from 30–150 nm of exosomes to 0.5–5 μm of the apoptotic bodies. Thus, they are certainly bionanomaterials and sometimes overlap with the definition of NPs given above when the three dimensions are all below 100 nm. Another close example is virus-like NPs, which are derived from the assembly of viral proteins into nanostructures with dimensions ranging from 20 to 500 nm for the delivery of various payloads [87]. So, besides physiological functions, EVs also have a direct role in pathological conditions such as cancer. Tumor cells secrete EVs to orchestrate intercellular communication with other tumor cells and stromal cells in the local and distant microenvironments. EVs play an essential role in both primary tumor growth and create the condition for metastasis formation, colonizing distant organs even before tumor cells and preparing tumor niche [88,89]. EVs orchestrate multiple systemic pathophysiological processes such as coagulation, vascular leakage, and reprogramming of recipient stromal cells to support pre-metastatic niche formation and subsequent metastasis [90]. Clinically, EVs may be biomarkers and novel therapeutic targets for cancer progression, particularly for predicting and preventing metastasis [91,92].

### 3.4. Nanomaterial and NPs Applications

Nanomaterials and NPs can be employed in medical practice for several purposes. The first application is their use for imaging when nanotools intrinsically possess optical properties, for instance, fluorescence, or are modified for the delivery of imaging agents [93,94]. These systems could reach the site of interest owing to their peculiar or acquired targeting ability, e.g., a surface equipped with an antibody toward the biomarker of a particular disease or reaction in a particular chemical condition [95], giving information about the progression of a pathology, or helping to perform an early diagnosis [96]. An example is the use of NPs with magnetic properties as contrast agents for magnetic resonance imaging. They indeed improve the quality, contrast, and amplification of the signal [97]. Additionally, due to the lower cytotoxicity compared with other agents such as Gd^3+^, they can be equipped with targeting moieties, shielded with coatings that preserve their properties and increment their shelf life, and even deliver therapeutic agents in a combination called “theranostic”, which verifies when NPs can both act as therapeutic and diagnostic items [98]. Theranostic NPs have known incredible success in recent years, not only in cancer treatment but also in other pathologies, such as Alzheimer’s disease, bacterial, and viral infection [99,100,101]. 

Another option is to use NPs as therapeutic agents. In this case, there are several possibilities: the first one is when NPs are directly exploited to cure the disease because they intrinsically possess peculiar properties that can contrast the pathology. An example is the use of NPs with selective toxicity toward bacteria, which treat the infection without affecting the surrounding tissues, such as the zinc oxide NPs [102]. In general, the strong bacterial toxicity of several types of NPs has been highlighted due to reactive oxygen species production and the release of ions, altering fundamental molecular pathways and the formation of biofilms while damaging cell membranes. Moreover, similar mechanisms of action have been reported for the damage of fungi [103]. Furthermore, some NPs, made, for example, by silver or zinc oxide, have demonstrated anti-viral properties [104,105]. Another strategy, widely studied for cancer treatment, is the use of NPs remotely activated by a second input, i.e., a physical stimulation, such as light or ultrasound. In this case, both NPs and the physical stimulation are given in a non-toxic dose because only their synergistic combination can affect cell viability. The rationale is that toxic effects are triggered only in the zone of interest where the two agents meet, leaving the neighbors safe. The mechanism of action is sometimes known; in other cases, it is under debate, but it generally depends on the nanomaterial and the stimulus involved. To maximize the therapeutic outcomes, the NPs are sometimes made with different materials, even responding to different inputs. In this specific case, the system is often deeply studied to achieve a theranostic combination [106]. There are several examples in the literature of NPs responsive to physical stimulations, such as gold, titanium dioxide, and zinc oxide NPs in combination with ultrasounds or shock waves. In the case of zinc oxide NPs, it has been demonstrated several times that they have an enhanced capability to cause cell death in combination with high energy shock waves in cervical adenocarcinoma and pancreatic cancer cells [107,108]. In both cases, it was evidenced that the cell death mechanism was related more to mechanical damage and zinc ion release than to the production of reactive oxygen species.

A third option is to use NPs for the delivery of therapeutics and/or imaging agents. In this case, the NPs could have a structure capable of being trapped inside the payload, such as liposomes, extracellular vesicles-derived materials that possess an internal hole, or a structure with pores, such as mesoporous silica [109]. Otherwise, the NPs should be chemically modified to expose, for instance, functional groups able to capture and transport the cargo [110].

**Figure 5:**
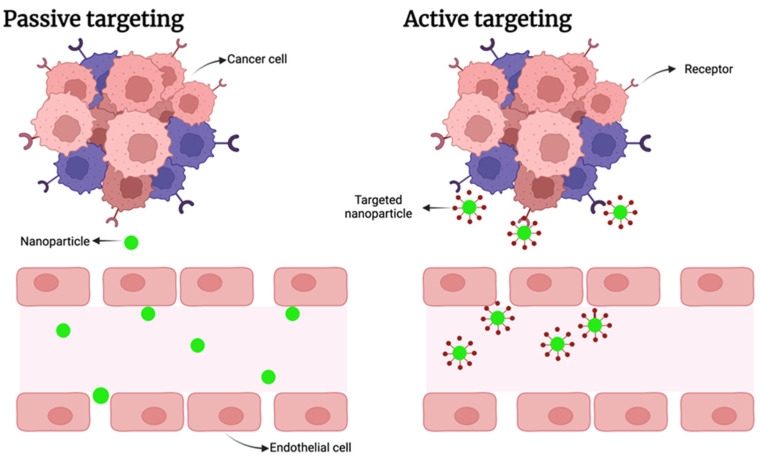
Differences between passive and active targeting. In the first case (on the left), naked NPs flow in the circulatory system and reach the tumor exploiting fenestrations in the vessels, but they are also distributed in other sites. In the active targeting (on the right), NPs are equipped with a targeting ligand, specifically recognized by the cancer cells; thus, they are captured preferably by these cells (created with Biorender.com).

In this context, drugs often represent the therapeutic agent delivered by NPs; indeed, this approach found a successful application for the treatment of several pathologies such as cardiovascular diseases, several bacteria-related infections, inflammatory bowel disease, and various cancers [111,112,113,114]. In the last case, in fact, NPs have been extensively investigated for anticancer drug delivery because they can preserve their cargo until reaching the target site, maximizing its efficacy and avoiding undesired off-target side effects. In addition, their insensitivity to some mechanisms responsible for multidrug resistance opens the possibility of novel therapeutic options for drug-resistant tumors. NPs could reach their target site by passive targeting, as in the case of cancer cells, where they exploit the enhanced permeabilization and retention effect, but are also able to penetrate several sites, such as the mucus layer, reaching the intestinal cells, and so on [115]. Another approach is to use active targeting, where the surface of the NPs is loaded with a ligand whose receptor is overexpressed by the target, such as the folate receptor or epidermal growth factor, which are present in several tumor types [116,117]. Both passive and active targeting are presented in Figure 5. Various drugs could be carried together [118].

NPs can also deliver agents responsive to stimuli, such as ultrasound and/or light. But for remotely activated ones, NPs generally do not interact with physical stimulation because their role is limited to the transport of the responsive agent. An example is the delivery of photo or sono-responsive molecules, for instance, porphyrins, to cancer cells. Exposure to light and/or ultrasound leads to the generation of reactive oxygen species that cause cell death [118]. NPs could also provide agents for immunotherapy. The main goal of immunotherapy is to activate the immune system against a target, such as tumor cells that develop immune escape mechanisms or a virus. A possibility is to use NPs carrying antigens directly to antigen-presenting cells that, in turn, activate the immune system, or for example, the delivery of checkpoint inhibitors [119]. NPs could also be used to deliver nucleic acids for gene therapy. It is theoretically able to resolve the pathology after a single administration, for example, by allowing for the expression or silencing of a specific gene and, thus, influencing the related pathway. Gene therapy is, therefore, proposed to treat several diseases, including cancer [120]. 

There are three cases: (i) editing through the CRISPR–Cas9 coding DNA plasmid or the Cas9 mRNA and single-guide RNA; (ii) gene addiction using DNA plasmid, minicircle DNA, mRNA, circular RNA, and self-amplifying RNA; (iii) gene downregulation with siRNA, ASOs, shRNA, and miRNA, among others [121].

Despite the enormous potential of gene therapy, there are several challenges in delivering nucleic acids to cells. For example, they are very unstable in body fluids, are subject to enzymes such as nucleases, and are rapidly cleared by the kidneys [122]. They also have difficulty penetrating cell membranes due to their negative charge, hydrophilic nature, and weight. Another problem could be their translocation into the nucleus. Various NPs have, thus, been proposed for delivering these molecules as an alternative to the use of viral-based gene delivery systems, which present some drawbacks. On the contrary, NPs could be designed to protect their payload by mediating their cytoplasmatic or nuclear delivery [123]. The latter is particularly challenging because the nuclear membrane is very selective and possesses small pores that discourage NP penetration. A strategy could lie in the addition of a molecular flag to the nucleic acid delivered, which mediates its nuclear translocation after its release in the cytosol [124]. Otherwise, some NPs with the ability to penetrate the nucleus have been proposed, e.g., coated with a nuclear-delivering peptide, but the size of the NPs is a crucial point [125]. For instance, Huo et al., exploring the size-dependent penetration ability of gold NPs, reported that only those of 2 nm and 6 nm diameters were found in the nucleus of MCF-7 breast cancer cells. Furthermore, they exploited 2 nm NPs for the nuclear delivery of a c-MYC binding triplex-forming oligonucleotide, successfully decreasing cell viability [125]. On the other hand, the delivery of molecules that must stay in the cytosol, e.g., miRNA and siRNA, is less challenging, and several NPs have been exploited for this purpose [126,127]. Interesting applications of miRNA delivered by NPs for the treatment of cancer are exhaustively described and reviewed elsewhere [128,129,130]. Moreover, a novel frontier of nucleic acid delivery by NPs is represented by oncogenic circRNA targeting or oncosuppresor circRNA delivery.

## 4. NP Delivery of circRNA Targeting Agents

The use of NPs in the context of circRNA targeting has a crucial advantage. NPs, indeed, effectively preserve their cargo, preventing its degradation and improving its specific delivery. Fervent research is ongoing in this direction, and Table 1 summarizes the examples collected so far, as circRNAs have been recently recognized as novel players with key biological roles in cancer. 

One of the first efficient studies of inorganic NPs promoting circRNA targeting as an antitumor strategy was performed on breast cancer models: gold NPs were used to directly target the circRNA *circDNMT1* or to interfere with its RBP-sponging properties. Since *circDNMT1* controls autophagy through the binding of Auf and p53 protein in breast cancer cells, both its direct silencing with specific siRNA and the vehiculation of specific ASOs interfering with the p53 and Auf binding sites on *circDNMT1* remarkably reduced tumor growth in vivo [44]. 

Gold NPs were also effective for the delivery of tumor-suppressor circRNAs or their expression vectors. Du et al. used gold NPs to overexpress circFoxo3 in melanoma tumor-bearing mice leading to tumor growth blockade [131]. Alternatively, Fang et al. used gold NPs to deliver ASOs that interfere with the binding sites of a circRNA derived from *cyclin B1* mRNA (*circCcnb1*) and Cdk1 proteins on *circCcnb1*, thereby increasing the overall survival of melanoma-bearing mice [132].

Another example of the use of more complex inorganic NPs for circRNA delivery comes from the study of So You et al. [133]. The authors developed siRNA-loaded superparamagnetic iron oxide NPs as a delivery tool in synergy with an external magnetic field to enhance the accumulation into the target tissue. The specific targeting of a circRNA derived from the *BRCA1 Associated RING Domain 1* mRNA (*circ_0058051*) in hepatocellular carcinoma cells and xenografts showed efficient antitumor activity [133]. Another successful targeting displaying antitumor effect in hepatocellular carcinoma was obtained with poly (β-amino esters) (PAEs) NP-mediated in vivo delivery of a siRNA against the circRNA derived from exon 5 of the *midkine* gene (circMDK) [134]. 

A further example is the NP delivery of the expression vector of the circRNA that originated from *Euchromatic Histone Lysine Methyltransferase 1* (*circEHMT1*). With this strategy, the breast cancer migration and invasion in vitro and the formation of metastasis in mice models were inhibited [135]. Furthermore, the intratumor injection with the Micropoly-transfectant reagent vehiculating the expression vector of the circRNA *0001073* (*circ-1073*) in a breast cancer model induced apoptosis [136]. Then, Shu et al. employed *Chitosan-epigallocatechin gallate* (*CS-EGCG*) NP-delivered overexpression plasmid for the circRNA derived from *Spire Type Actin Nucleation Factor 1* (*circSPIRE1*) to suppress renal cancer metastasis in mice. This circRNA forms a complex with the ELAV-like RNA binding protein 1 (ELAVL1) and *acetylgalactosaminyltransferase 3* (*GALNT3*) mRNA enhancing the epithelial state through E-cadherin glycosylation, and suppressing cell migration, proliferation, and angiogenesis [137]. Another study developing NP-delivered circRNA targeting was performed for the treatment of gastric cancer. In this context, a cirRNA derived from the *Nuclear Receptor Interacting Protein 1* (*circNRIP1*) acts as a competing endogenous RNA sponging the *miR-149-5p*. Zhang et al. proposed a transient silencing of *circNRIP1* using an organic cholesterol NP to deliver a specific siRNA [138]. Moreover, Zhou et al. developed a poly lactic-co-glycolic acid (PLGA)-based NP loaded with a siRNA targeting the back-splicing junction of *cSERPINE2*. This circRNA is overexpressed in breast cancer and controls the secretion of Interleukin-6 sustaining proliferation and invasion of cancer cells [139]. A further example of NP-mediated siRNA delivery comes from Meng et al.’s study, in which siRNA loaded into PLGA-based NP was used to specifically silence *circROBO1*. This circRNA is involved in hepatocellular carcinoma progression controlling the *miR-130a-5p*/CCNT2 axis [140].

Moreover, NPs are used for the delivery of synthetic circRNAs to contrast the action of miRNAs, such as *miR-21-5p*, which is abundant in cancer. Indeed, Müller et al. used polyethyleneimine-based NPs for the delivery of circRNA to decoy *miR-21-5p* (*miR-21-5p ciRs*) in lung adenocarcinoma cells and tumor models. It was shown that this treatment impairs the oncogenic potential in vitro and tumor growth in vivo [141]. 

Finally, since exosomes represent an even more natural and exploitable NP strategy, Wang et al. [142] modified active exosomes by introducing a specific siRNA targeting *ciRS-122* and obtained the restoration of sensitivity to oxaliplatin in in vivo models. 

**Table 1:** Summary of NP-delivered targeting of cancer-related circRNAs, their molecular mechanism, and biological effects.

Cancer	NPs	CircRNA	Molecular Mechanism	Antitumor Activity	Ref.
Breast	Gold NPs/siRNA or ASO	circDNMT1	p53 transcription inhibition	Autophagy suppression	[44]
Melanoma	Gold NPs/circRNA	circFoxo3	Interaction with MDM2/p53	Apoptosis induction	[131]
Melanoma	Gold NPs/ASO	circCcnb1	Interaction with Ccnb1/Cdk1	Cell invasion suppression	[131]
Hepatocellular carcinoma	Superparamagnetic iron oxide NPs/siRNA	circ_0058051	P38 downregulation	Migration/proliferation inhibition	[133]
Hepatocellular carcinoma	PAEs NPs/siRNA	circMDK	PI3K/AKT/mTOR pathway modulation	Proliferation/invasion/migration inhibition	[134]
Breast	Gold NPs/expression vector	circEHMT1	MMP2 pathway modulation	Migration/invasion inhibition	[134]
Breast	Micropoly-Transfecter NPs/expression vector	circ-1073	Cleaved Caspase-3/9 overexpression	Apoptosis induction	[135]
Renal cell carcinoma	CS-EGCG NPs/expression vector	circSPIRE1	E-cadherin glycosylation	Proliferation/angiogenesis/metastasis suppression	[137]
Gastric	Organic cholesterol NPs/siRNA	circNRIP1	miR-149-5p sponging	Chemotherapy sensitivity restoration	[138]
Breast	PLGA-NPs/siRNA	cSERPINE2	MALT1-NF-*κ*B-IL-6 axis	Proliferation/migration inhibition	[139]
Hepatocellular carcinoma	PLGA-NPs/siRNA	circROBO1	miR-130a-5p/CCNT2 axis modulation	Proliferation/invasion inhibition	[140]
Lung adenocarcinoma	Polyethyleneimine-based NPs/circRNA	miR-21-5p ciRs	miR-21-5p decoy	Proliferation/invasion inhibition	[140]
Colorectal	Exosomes/siRNA	ciRS-122	PKM2 downregulation	Chemotherapy sensitivity restoration	[142]

Apart from cancer, NPs delivering agents silencing or overexpressing circRNAs have been proposed for the treatment of other pathologies, such as cardiac diseases [141,142,143] and their related consequences, and for facilitating wound repair. Yang et al. demonstrated that the use of gold NPs for the delivery of *circAmotl1* can improve cardiac functions in mice models of doxorubicin-induced cardiomyopathy, promoting cell proliferation, adhesion, and migration and, thereby, accelerating wound repair [143]. NP-based circRNA targeting also represents a promising strategy for the treatment of acute ischemic stroke (AIS). Liu et al. showed that *CircOGDH* was significantly upregulated in the plasma of AIS patients. Using PLGA-based NPs, *CircOGDH* siRNA was delivered in the middle cerebral artery occlusion/reperfusion (MCAO/R) of mouse models, and apoptosis of neurons into the ischemic penumbra region was reduced [144]. These studies highlight the potential of NPs targeting circRNAs as a novel therapeutic approach in cancers and other pathological conditions.

## 5. Conclusions

Nowadays, circRNAs are under intensive investigation for their role in cancer and other pathologies. Significant efforts have been made to validate circRNAs’ applicability as therapeutic targets or therapeutic agents. Moreover, advancements in circRNA research have led to the development of innovative delivery systems, including NP-based carriers, which enhance the stability, cellular uptake, and targeted distribution of their cargos. However, while NPs offer a promising approach to circRNA delivery, several limitations need to be addressed to fully realize their therapeutic potential, e.g., ameliorating their biodistribution, pharmacokinetics, long-term safety, and overcoming hurdles in material synthesis, design, and manufacturing process. Even bio-derived materials, such as EVs, present some unresolved questions that limit their usage. Indeed, large-scale industrial production and final output standardization must be reached. Therefore, careful consideration of NPs properties remains crucial to advancing their efficacy and applicability in biomedical applications. Fervent work in this direction and the increasing number of findings regarding the role of circRNAs will pave the way for the specific and efficient delivery of key novel antitumor agents.
